# Disparities in exclusive breastfeeding by current maternal employment status in Peru, 2005–2023: a cross-sectional analysis of ENDES data

**DOI:** 10.3389/fgwh.2026.1865825

**Published:** 2026-07-20

**Authors:** Lily Mabel Portal-Valqui, Kevin Cusma-Regalado, Jhofree Einstein Briceño-Chavez, Juan Carlos Bustamante-Rodríguez, Jhosmer Ballena-Caicedo, Fiorella E. Zuzunaga-Montoya, Víctor Juan Vera-Ponce

**Affiliations:** Facultad de Medicina (FAMED), Universidad Nacional Toribio Rodríguez de Mendoza de Amazonas (UNTRM), Chachapoyas, Peru

**Keywords:** exclusive breastfeeding, health inequalities, maternal employment, Peru, women's work

## Abstract

**Introduction:**

Exclusive breastfeeding (EBF) improves child survival and maternal health, but structural and employment-related conditions may affect its continuity. Evidence from Peru on EBF disparities by current maternal employment status remains limited.

**Objective:**

To estimate descriptive disparities in EBF prevalence by current maternal employment status and describe their temporal and territorial patterns in Peru from 2005 to 2023.

**Methods:**

We conducted an analytical cross-sectional study using pooled data from the Peruvian Demographic and Family Health Survey [Encuesta Demográfica y de Salud Familiar (ENDES)] from 2005 to 2023. The revised descriptive sample included 15,780 mother-child dyads with infants younger than six months and mothers currently married or cohabiting. EBF was defined by 24 h recall. Maternal employment status was classified as no current survey-defined employment, current active formal-sector employment, or current active informal-sector employment, based on current work and employer type. Survey-weighted prevalences and crude and adjusted prevalence ratios (PRs) were estimated; adjusted models included maternal age, educational level, wealth quintile, parity, child age, child sex, residence area, natural region, and survey year. Temporal trends, absolute gaps, and departmental ecological correlations were assessed.

**Results:**

The revised weighted prevalence of EBF was 72.6%. In the complete-case model sample (*n* = 15,101), EBF prevalence was 76.2% among mothers with no current survey-defined employment, 46.5% among mothers with current active formal-sector employment, and 74.2% among mothers with current active informal-sector employment. Compared with no current survey-defined employment, current active formal-sector employment was associated with lower EBF prevalence in both crude (PR = 0.61; 95% CI: 0.55–0.67) and adjusted models (adjusted PR = 0.72; 95% CI: 0.65–0.79). Current active informal-sector employment showed little difference after adjustment (adjusted PR = 0.97; 95% CI: 0.94–1.00). At the departmental level, current active formal-sector employment was negatively correlated with EBF prevalence (*r* = −0.72; *p* < 0.001).

**Conclusions:**

In this descriptive cross-sectional analysis, current active formal-sector employment was associated with lower EBF prevalence. These findings identify mothers actively participating in formal-sector work as a group requiring further evaluation of employment-compatible breastfeeding support, without implying causal effects of labor formality or direct evidence about specific workplace or health policies.

## 1 Introduction

Exclusive breastfeeding (EBF) is defined as feeding an infant only breast milk during the first six months of life, without other liquids or foods except indicated medicines, vitamins, or minerals. This practice is a key public health intervention associated with reduced infant morbidity and mortality and sustained benefits for maternal health (1, 2). Despite these recommendations, EBF remains insufficient in many regions of the world: the World Health Organization estimates that approximately 44% of infants aged 0 to 6 months receive EBF, whereas the Global Breastfeeding Scorecard 2023 reported a global prevalence close to 48%, still below international targets for sustained improvement (3, 4).

Beyond its biological importance, breastfeeding is a social phenomenon shaped by structural determinants. Evidence shows that breastfeeding practices are not uniformly distributed and may reflect socioeconomic, territorial, and labor-related inequalities (5, 6). Evidence from maternity-leave analyses and workplace-intervention reviews suggests that return-to-work timing, protected breaks, private spaces for milk expression, storage facilities, and supervisor support are relevant conditions for maintaining breastfeeding among employed mothers (6–8). This issue is especially relevant in contexts where different labor regimes and degrees of social protection coexist, with differences in stability, labor rights, and effective access to benefits.

In Latin America, population-based surveys have documented important variations in EBF prevalence across countries and population groups. In Chile, the National Breastfeeding Survey in Primary Care reported that 56.3% of mothers achieved EBF for six months or more (9). In Peru, ENDES-based studies have described socioeconomic and territorial inequalities in EBF, with higher prevalence reported among infants from lower-wealth households, rural areas, and the Highlands and Amazon regions, and lower prevalence in urban settings, Metropolitan Lima, and higher-wealth groups (10). However, studies analyzing the relationship between current maternal employment status, employer type, and EBF practices in the country remain scarce.

To address this evidence gap, the present study aimed to estimate descriptive disparities in EBF prevalence according to current maternal employment status among mothers currently married or cohabiting with children younger than six months in Peru, using data from nineteen ENDES rounds corresponding to 2005–2023. The study aims to provide evidence for identifying groups requiring employment-compatible breastfeeding support, while recognizing that the exposure represents current participation in formal-sector work rather than administrative labor formality itself.

## 2 Methods

### 2.1 Study design

We conducted an observational analytical cross-sectional study based on repeated surveys, using publicly available secondary data from the Peruvian Demographic and Family Health Survey (ENDES). The analysis included 19 consecutive annual rounds corresponding to 2005–2023. The analytical objective was to estimate absolute and relative disparities in EBF prevalence according to current maternal employment status, operationally classified as no current survey-defined employment, current active formal-sector employment, and current active informal-sector employment. The study was reported in accordance with the STROBE guideline for observational cross-sectional studies (11).

### 2.2 Data source

ENDES is a continuous population-based survey conducted by the National Institute of Statistics and Informatics (INEI), designed to generate information on demographic dynamics, maternal and child health, infant feeding practices, household characteristics, access to services, and sociodemographic conditions of the Peruvian population (12). The survey includes structured interviews administered to women of reproductive age who are usual household residents, as well as specific modules on births, breastfeeding, child feeding, maternal and child health, and employment status.

ENDES uses a complex, probabilistic, stratified, two-stage sampling design with inference at the national, urban-rural, natural-region, and departmental levels. In the first stage, clusters are selected with probability proportional to size; in the second stage, dwellings are selected within each cluster. The design is independent by study domain, and each annual round has its own expansion factors, strata, and primary sampling units. The datasets and technical documentation for each round are publicly available through the INEI institutional portal (12).

### 2.3 Population and sample

The source population consisted of women aged 15 to 49 years interviewed in ENDES between 2005 and 2023 who had birth records available in the corresponding module. The unit of analysis was the mother-child dyad. The primary analytical sample included dyads meeting the following criteria: mother interviewed in one of the included rounds; child younger than six months at the time of the interview; information available in the breastfeeding and infant feeding module; mother currently married or cohabiting; and sufficient information to define the outcome and critical analysis variables. Stratified analyses by employment status excluded records without a valid employment classification.

Records of children aged six months or older were excluded because the standard EBF indicator is defined for infants younger than six months. Mothers not currently married or cohabiting, including single, separated, divorced, or widowed women, were excluded from the primary analysis to preserve the prespecified analytical population; however, because marital status may be associated with employment, household support, breastfeeding continuation, and socioeconomic conditions, we conducted a sensitivity analysis including all mothers regardless of marital status. Finally, a complete-case analysis was applied; no missing-data imputations were performed. As a result, inference from the primary analysis corresponds to mothers currently married or cohabiting with infants younger than six months and does not necessarily extend to all women with children of that age in the country.

### 2.4 Variable processing and harmonization

Before analysis, annual datasets were harmonized to ensure temporal comparability. The survey year was obtained from the year variable available in the consolidated dataset and, when necessary, completed using the ENDES interview-date variable; values recorded in two-digit format were standardized to four-digit calendar years. Child age in months was derived from the infant age variable reported in the survey and was used both to restrict the sample to children younger than six months and to construct descriptive age categories.

The current breastfeeding indicator was harmonized by combining equivalent variables from the breastfeeding module available across rounds, so that minor changes in annual coding would not produce artificial loss of information. Similarly, employment status, education, parity, wealth, residence area, and natural region variables were recoded into homogeneous and mutually exclusive categories before pooling the rounds. Values coded as “do not know”, “does not remember”, “not applicable”, or inconsistent with the analytical definition were treated as missing when appropriate.

### 2.5 Variables

The main outcome was exclusive breastfeeding. EBF was operationalized using the standard 24-hour recall indicator: an infant younger than six months who was still receiving breast milk at the time of the interview and who, during the previous 24 h, had not received water, infant formula, other milk, other liquids, or semisolid or solid foods, except medicines, vitamins, or minerals (13). Operationally, children meeting all of these conditions were classified as EBF = 1, and those who did not were classified as EBF = 0. This definition estimates EBF practice on the day before the survey and therefore should not be interpreted as uninterrupted EBF continuity throughout the period from birth to six months.

The main exposure was current maternal employment status at the time of the interview. Mothers who did not report current survey-defined employment were classified as having no current survey-defined employment; this label does not imply absence of unpaid household or caregiving work. Among mothers reporting current work, current active formal-sector employment was assigned to those working for the government or a private company. Current active informal-sector employment was assigned to those reporting self-employment, unpaid family work, or other occupational modalities. Records without a valid employment category were excluded from analyses stratified by employment status.

This classification should be interpreted as a pragmatic proxy based on current work and employer type, not as an administrative measure of labor formality. Therefore, self-employed women operating in the formal sector may have been misclassified as informal, and women with a formal job who were temporarily away from work because of maternity leave or another reason may have been classified as having no current survey-defined employment. Consequently, the formal-sector category is best interpreted as active participation in formal-sector work at the time of survey and may partly capture early postpartum return to formal-sector work.

Covariates were also considered. Maternal, child, and household characteristics were included because of their epidemiological relevance and their potential relationship with breastfeeding practices. Maternal age was obtained in completed years and categorized as 15–19, 20–34, and 35–49 years. Educational attainment was derived from the highest level completed and grouped as no education/initial, primary, secondary, and higher education. Parity was constructed from the total number of live-born children and classified as primiparous, 2–3 children, and 4 or more children. Child sex was obtained from the births module. Child age was grouped as 0 ≤ 2, 2 ≤ 4, and 4 ≤ 6 months for descriptive presentation of the age gradient in EBF.

The wealth quintile corresponded to the asset index provided by ENDES, constructed by INEI from household assets and characteristics using principal component analysis; Q1 represents the lowest wealth quintile and Q5 the highest. Residence area was classified as urban or rural. Natural region was grouped into Metropolitan Lima, Rest of Coast, Highlands, and Amazon according to the territorial coding available in the survey. For the exploratory interaction analysis, socioeconomic level was dichotomized as not high (Q1–Q3) and high (Q4–Q5), and residence area as rural or urban.

Mode of delivery and place of delivery were evaluated in a sensitivity analysis because they may be associated with breastfeeding and healthcare access. Breastfeeding-counselling variables were not included in the primary model because comparable information was not consistently available across all 2005–2023 survey rounds and missingness followed a year-dependent pattern. Their omission may leave residual confounding; therefore, these variables were treated as limitations rather than as primary adjustment covariates.

Complementarily, we analyzed the reported reason for breastfeeding cessation among mothers whose youngest child was no longer receiving breast milk at the time of the interview. This variable was obtained from the ENDES breastfeeding module. The categories reported by the survey were retained for descriptive analysis, excluding non-informative responses such as do not know or does not remember. This evaluation was considered exploratory because it applies only to mothers who had already stopped breastfeeding and reflects a single reported main reason, not necessarily the full set of factors that led to cessation.

### 2.6 Statistical analysis

Analyses incorporated the complex ENDES sampling design through weighting factors, strata, and primary sampling units. For the pooled analysis of the 19 annual rounds, each year's sampling weight was rescaled by dividing it by the number of rounds included, to avoid having the combined file represent the accumulated population size across all years. Thus, the pooled estimate corresponded to an average prevalence for 2005–2023, not to an accumulated population total.

First, the analytical sample was described according to maternal employment status. Categorical variables were summarized using unweighted absolute frequencies and weighted percentages, incorporating the sampling design. Next, the weighted prevalence of EBF was estimated with 95% confidence intervals (95% CIs) overall and according to categories of employment status, maternal characteristics, child characteristics, and household variables.

The association between current maternal employment status and EBF was evaluated using survey-weighted Poisson regression models with a log link and robust standard errors, reporting crude and adjusted prevalence ratios (PRs) with 95% CIs (14). This approach was chosen because EBF was a frequent outcome and, in this context, odds ratios from logistic models may overestimate the magnitude of association relative to prevalence ratios. The reference group was mothers with no current survey-defined employment. Crude PRs were interpreted as descriptive measures of total relative disparity. Minimally adjusted models included maternal age, educational level, wealth quintile, parity, child age, child sex, urban/rural residence, natural region, and survey year as a categorical covariate. Adjusted PRs were interpreted as sensitivity estimates assessing the robustness of the employment-status disparity after accounting for major compositional differences, not as estimates of a causal effect.

A sensitivity analysis included all mothers with infants younger than six months, regardless of marital status, and additionally adjusted for marital status. A second sensitivity analysis added mode of delivery and place of delivery to the main adjusted model. Variables on breastfeeding counselling were not included in the primary or sensitivity models because harmonized and comparable information was not available across all survey rounds.

Population attributable fractions were not estimated because the study did not specify a formal causal counterfactual and the main exposure was an operational proxy for current employment status. Interpretation focused on weighted prevalences, absolute gaps, and crude PRs as descriptive measures of disparity.

**Figure 1:**
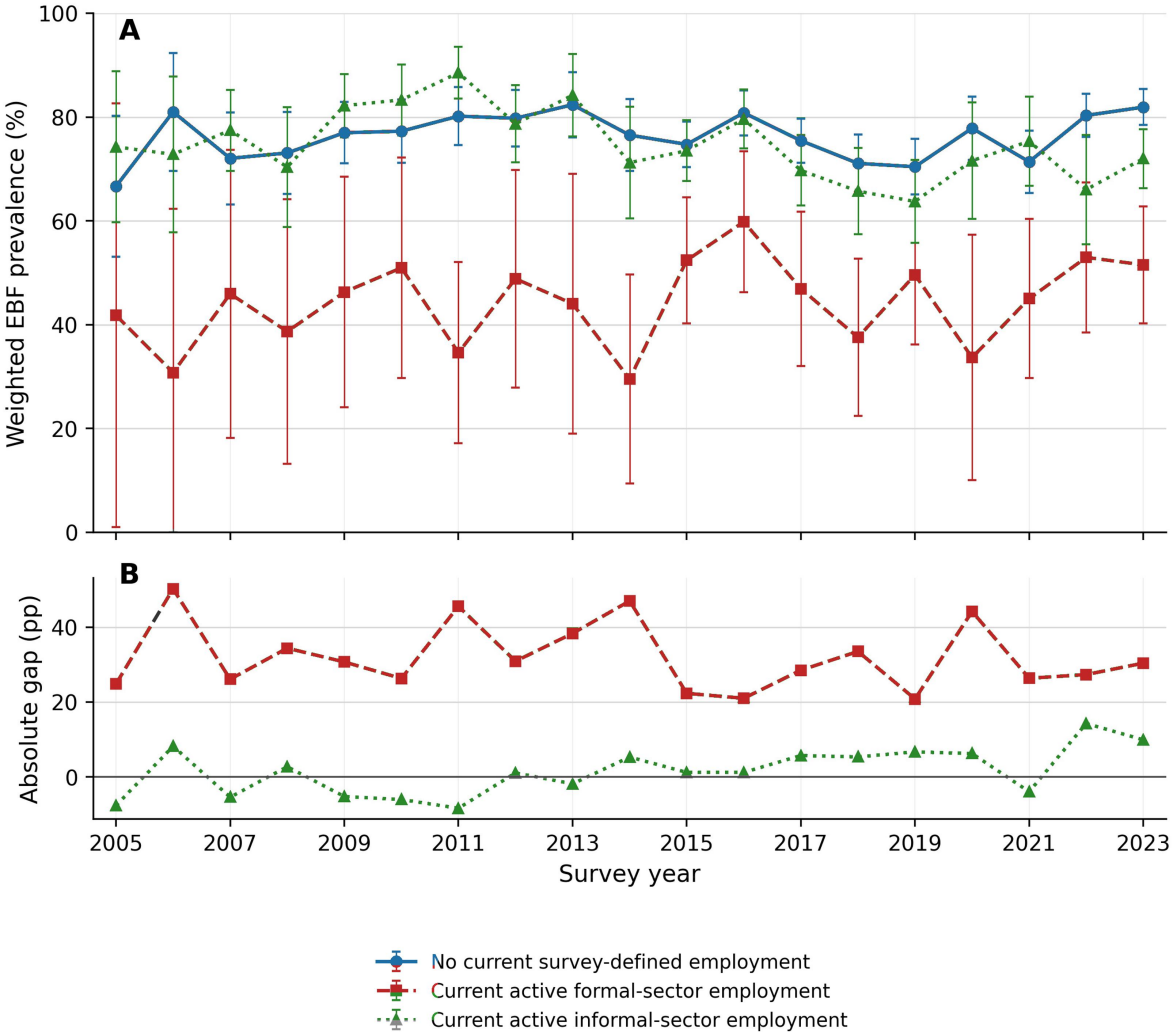
Weighted annual exclusive breastfeeding prevalence according to current maternal employment status (Panel **A**) and evolution of the EBF gap between employment groups (Panel **B**). Peru, ENDES 2005–2023. Panel **A**: survey-weighted annual prevalences with 95% CIs accounting for the complex ENDES sampling design. Panel **B**: absolute gap (percentage points) between the EBF prevalence of mothers with no current survey-defined employment and each group of workers. Estimates are descriptive and do not correspond to a causal evaluation of public policy. Sample sizes for current active formal-sector employment were small in some early years, resulting in wider confidence intervals.

For the temporal analysis, annual EBF prevalences according to current maternal employment status were estimated for 2005–2023, and absolute gaps between mothers with no current survey-defined employment and each group of workers were calculated in percentage points. Figure 1 was regenerated using weighted annual estimates and 95% CIs to maintain consistency with the complex ENDES sampling design. Temporal heterogeneity was assessed using interaction terms between employment status and survey year as a continuous variable and between employment status and prespecified periods (2005–2012, 2013–2019, 2020–2021, and 2022–2023). Period-specific adjusted PRs were estimated using the same covariate set as the main adjusted model, excluding survey year when period was used as the stratifying variable. These analyses had a descriptive purpose related to temporal stability and did not constitute a causal evaluation of regulatory changes or public policies.

Reasons for breastfeeding cessation were summarized using weighted proportions with 95% CIs, stratified by maternal employment status. Standard errors were estimated accounting for the complex sampling design through Taylor linearization. This analysis was restricted to mothers with valid information on breastfeeding cessation and was considered exploratory.

For the territorial analysis, weighted prevalences of EBF and weighted proportions of maternal current active formal-sector employment were estimated for 25 political-administrative units in Peru, corresponding to the 24 departments and the Constitutional Province of Callao. These estimates were used to produce choropleth maps and to assess the ecological association between both variables using Pearson's correlation coefficient. This analysis was interpreted at the aggregate territorial level; therefore, it was not used to infer individual associations or causal mechanisms.

Finally, an exploratory additive interaction analysis was conducted between current active formal-sector employment and high socioeconomic level, as well as between current active formal-sector employment and urban residence. For this analysis, the employment exposure was dichotomized as current active formal-sector employment vs. non-formal current employment status, with the latter category grouping mothers with no current survey-defined employment and those with current active informal-sector employment. Four-category combined variables were constructed for each pair of exposures, and crude PRs were estimated using Poisson regression with the survey design. From these PRs, the relative excess risk due to interaction (RERI), the attributable proportion due to interaction (AP), and the synergy index (S) were calculated, with 95% CIs obtained using the delta method through the nlcom command in Stata, following recommendations for presenting interaction analyses (15). Because EBF is a favorable outcome, these measures were interpreted cautiously as deviations from additivity in EBF prevalence, not as definitive evidence of causal interaction or as quantification of harm.

All analyses were performed in Stata version 17 (StataCorp, College Station, TX, USA). A *p* value <0.05 was considered the criterion for statistical significance in inferential analyses; however, interpretation prioritized the magnitude, direction, and consistency of the estimates, in line with the descriptive objective of disparities.

### 2.7 Ethical considerations

The study used exclusively anonymized, publicly available secondary microdata from ENDES, available through INEI. At no stage of the analysis was information accessed that could identify individual participants or their children. Because this was a secondary analysis of anonymized public datasets, with no intervention involving individuals and no primary data collection, additional approval by an institutional ethics committee was not required. The original ENDES data collection is conducted under INEI institutional protocols, with informed consent and confidentiality safeguards for participants.

## 3 Results

### 3.1 Sample selection

After harmonization and restriction to the 2005–2023 ENDES rounds, 18,882 mother-child dyads with infants younger than six months were identified. We excluded 2,919 dyads because the mother was not currently married or cohabiting or marital status was unavailable, leaving 15,963 dyads before complete-case exclusions. After excluding 183 observations with critical missing information in variables required for the descriptive analysis, the restricted descriptive sample consisted of 15,780 mother-child dyads. Employment-stratified descriptive analyses excluded seven records without a valid employment classification, and the complete-case sample for the adjusted model included 15,101 dyads. The study population selection process is shown in Supplementary Figure S1.

### 3.2 Characteristics of the study population

The descriptive sample included 15,780 mother-child dyads with infants younger than six months. Among mothers with valid employment information for classification (*n* = 15,773), 59.2% had no current survey-defined employment, 31.2% had current active informal-sector employment, and 9.7% had current active formal-sector employment. Regarding maternal age, the largest proportion of women was in the 20–34-year age range, representing 65.7% of mothers with no current survey-defined employment, 62.1% of mothers with current active informal-sector employment, and 63.9% of mothers with current active formal-sector employment. Mothers with current active formal-sector employment had a higher proportion of women aged 35–49 years (32.1%) than mothers with no current survey-defined employment (18.3%) and current active informal-sector employment (20.4%).

With respect to socioeconomic characteristics, 50.5% of mothers with current active formal-sector employment belonged to the highest wealth quintile, whereas 24.5% of mothers with no current survey-defined employment were in the lowest quintile. Higher education was more frequent among mothers with current active formal-sector employment (46.0%). Regarding residence area, 93.9% of mothers with current active formal-sector employment lived in urban areas, with Metropolitan Lima accounting for 46.2% of this group (Table 1).

**Table 1:** Sample characteristics according to current maternal employment status.

Characteristic	Total (*n* = 15,780)	No current survey-defined employment (*n* = 9,423; 59.2%)	Current active formal-sector employment (*n* = 1,259; 9.7%)	Current active informal-sector employment (*n* = 5,091; 31.2%)
Maternal age (years)				
15–19	2,272 (15.3)	1,378 (16.0)	60 (4.1)	833 (17.5)
20–34	10,156 (64.4)	6,201 (65.7)	815 (63.9)	3,134 (62.1)
35–49	2,829 (20.3)	1,513 (18.3)	358 (32.1)	958 (20.4)
Educational level				
No education/initial	5,519 (33.7)	3,379 (34.3)	399 (30.2)	1,741 (33.8)
Primary	3,625 (21.5)	2,178 (22.6)	141 (9.8)	1,301 (23.0)
Secondary	4,419 (28.7)	2,750 (30.4)	174 (14.0)	1,493 (29.9)
Higher education	2,216 (16.1)	1,115 (12.8)	545 (46.0)	556 (13.2)
Parity				
Primiparous	3,148 (22.7)	2,026 (24.0)	387 (33.6)	730 (16.9)
2–3 children	8,176 (52.4)	4,958 (52.5)	765 (60.1)	2,451 (50.1)
4 or more children	4,306 (24.8)	2,362 (23.6)	90 (6.3)	1,854 (33.0)
Child sex				
Male	8,084 (51.7)	4,862 (51.7)	641 (52.2)	2,580 (51.5)
Female	7,696 (48.3)	4,561 (48.3)	618 (47.8)	2,511 (48.5)
Child age (months)				
0 ≤ 2	3,604 (22.5)	2,408 (24.9)	250 (20.3)	939 (18.6)
2 ≤ 4	5,660 (35.6)	3,386 (35.8)	438 (34.5)	1,836 (35.6)
4 ≤ 6	6,516 (41.9)	3,629 (39.4)	571 (45.2)	2,316 (45.8)
Wealth quintile				
Q1 (poorest)	4,774 (22.8)	2,992 (24.5)	45 (2.0)	1,737 (26.1)
Q2	4,349 (24.3)	2,795 (26.6)	166 (8.5)	1,383 (24.7)
Q3	3,085 (20.8)	1,907 (22.5)	225 (13.6)	951 (19.6)
Q4	2,134 (17.3)	1,116 (15.6)	358 (25.5)	660 (17.8)
Q5 (richest)	1,438 (14.9)	613 (10.7)	465 (50.5)	360 (11.8)
Residence area				
Urban	9,810 (70.5)	5,836 (70.2)	1,122 (93.9)	2,850 (64.0)
Rural	5,970 (29.5)	3,587 (29.8)	137 (6.1)	2,241 (36.0)
Natural region				
Metropolitan Lima	1,521 (28.4)	887 (27.4)	216 (46.2)	418 (24.8)
Rest of Coast	4,246 (25.2)	2,703 (27.2)	415 (25.9)	1,126 (21.2)
Highlands	5,525 (29.3)	3,040 (27.6)	347 (18.8)	2,133 (35.7)
Amazon	4,488 (17.0)	2,793 (17.7)	281 (9.1)	1,414 (18.2)

Values are expressed as *n* (weighted %). Percentages correspond to weighted estimates accounting for the complex ENDES sampling design. Employment categories sum to 15,773 because seven records lacked a valid employment classification. Missing data for the restricted descriptive sample were mainly maternal age (*n* = 523), parity (*n* = 150), and employment classification (*n* = 7).

Peru, ENDES 2005–2023 (*N* = 15,780).

### 3.3 EBF prevalence according to current maternal employment status

The overall weighted prevalence of EBF was 72.6%. EBF was more frequent among mothers with no current survey-defined employment (76.1%) and current active informal-sector employment (74.1%) than among mothers with current active formal-sector employment (46.8%). In addition, it decreased with maternal age, from 75.7% among adolescents aged 15–19 years to 68.9% among women aged 35–49 years, and it was lower among mothers with higher education (62.1%) than among those with primary education (77.5%), secondary education (73.3%), or no education/initial education (74.0%).

EBF was more frequent among mothers with four or more children (78.7%) than among primiparous mothers (65.0%). According to child characteristics, it was slightly higher among girls (73.0%) than among boys (72.3%) and declined with infant age, from 79.5% at 0 ≤ 2 months to 66.9% at 4 ≤ 6 months. Socioeconomically, EBF was higher in the poorest quintile (86.6%) than in the richest quintile (51.1%). It was also higher in rural areas (84.9%) than in urban areas (67.5%) and reached its highest values in the Highlands (82.2%) and Amazon region (80.3%), whereas Metropolitan Lima recorded the lowest value (62.9%) (Supplementary Table S1).

### 3.4 Association between current maternal employment status and EBF

In the restricted complete-case sample for the adjusted model (*n* = 15,101), current active formal-sector employment was associated with lower EBF prevalence compared with no current survey-defined employment in both crude and adjusted analyses (crude PR = 0.61; 95% CI: 0.55–0.67; adjusted PR = 0.72; 95% CI: 0.65–0.79). Current active informal-sector employment showed little difference after adjustment (crude PR = 0.97; 95% CI: 0.94–1.01; adjusted PR = 0.97; 95% CI: 0.94–1.00) (Table 2).

**Table 2:** Crude and adjusted prevalence ratios for exclusive breastfeeding according to current maternal employment status.

Current maternal employment status	*n*/*N*	EBF prevalence (%; 95% CI)	Crude PR (95% CI)	Adjusted PR (95% CI)
No current survey-defined employment	7,016/9,016	76.2 (74.8–77.5)	Reference	Reference
Current active formal-sector employment	603/1,216	46.5 (42.0–50.9)	0.61 (0.55–0.67)	0.72 (0.65–0.79)
Current active informal-sector employment	3,709/4,869	74.2 (72.2–76.2)	0.97 (0.94–1.01)	0.97 (0.94–1.00)

EBF, exclusive breastfeeding; PR, prevalence ratio.

Prevalences and 95% CIs come from estimates weighted by the complex sampling design. PRs were estimated using survey-weighted Poisson regression with log link and robust variance, using no current survey-defined employment as the reference. The adjusted model included maternal age, educational level, wealth quintile, parity, child age, child sex, urban/rural residence, natural region, and survey year as a categorical covariate. Estimates are interpreted as descriptive and robustness measures, not as causal effects.

Peru, ENDES 2005–2023.

The sensitivity analysis including all mothers with infants younger than six months and additionally adjusting for marital status yielded similar results for current active formal-sector employment (adjusted PR = 0.74; 95% CI: 0.68–0.81). Additional adjustment for mode of delivery and place of delivery did not materially change the estimate for current active formal-sector employment (adjusted PR = 0.73; 95% CI: 0.66–0.81) (Supplementary Tables S4, S6).

### 3.5 Temporal trends and absolute EBF gaps

Temporal trends in weighted EBF prevalence according to current maternal employment status during 2005–2023 are shown in Figure 1A. In most years, mothers with no current survey-defined employment and those with current active informal-sector employment had similar prevalences, both higher than those observed among mothers with current active formal-sector employment. Interaction testing showed some evidence of temporal heterogeneity when survey year was modeled continuously (*p* = 0.041), whereas the interaction by prespecified period was suggestive but not conventionally significant (*p* = 0.058). The inverse association for current active formal-sector employment persisted across all prespecified periods, with adjusted PRs ranging from 0.63 to 0.76 (Supplementary Table S5).

The annual absolute gap in percentage points between mothers with no current survey-defined employment and current active formal-sector workers, as well as between mothers with no current survey-defined employment and current active informal-sector workers, is shown in Figure 1B. Across the period analyzed, the gap was larger between mothers with no current survey-defined employment and current active formal-sector workers than between mothers with no current survey-defined employment and current active informal-sector workers. This time series should be interpreted as an exploratory description based on weighted annual estimates; the current active formal-sector subgroup was small in some early years, leading to wider confidence intervals.

### 3.6 Reasons for breastfeeding cessation according to current maternal employment status

Among mothers who reported breastfeeding cessation (*n* = 4,049), the most frequent reasons were weaning age (31.6%) and becoming pregnant (24.3%). Other reported reasons included insufficient breast milk (10.6%), another health reason (8.9%), work (8.1%), and no breast milk (8.0%).

According to current maternal employment status, the reason “work” was reported by 18.1% of mothers with current active formal-sector employment, compared with 7.5% of mothers with current active informal-sector employment and 6.5% of mothers with no current survey-defined employment. The reason “no breast milk” was reported by 17.8% of mothers with current active formal-sector employment, compared with 6.3% of mothers with current active informal-sector employment and 7.0% of mothers with no current survey-defined employment. These two reasons were presented separately because “work” directly reflects an occupational constraint, whereas “no breast milk” may reflect perceived milk insufficiency, physiological concerns, counselling gaps, or social beliefs. Therefore, the cessation-reason analysis was interpreted descriptively and not as evidence of a single mechanism (Supplementary Table S2).

### 3.7 Territorial distribution and ecological correlation between maternal current active formal-sector employment and EBF

**Figure 2:**
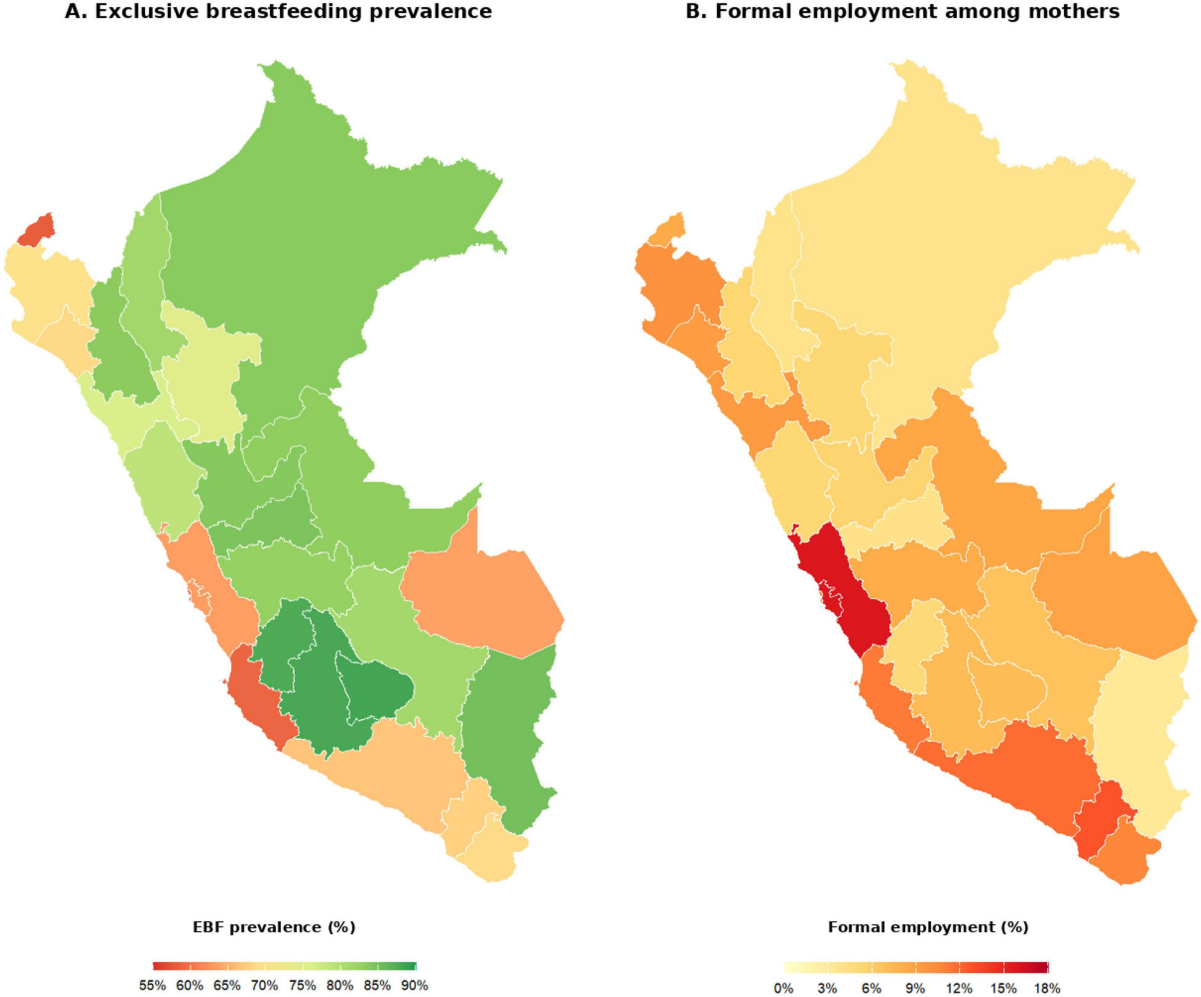
Geographic distribution of exclusive breastfeeding and maternal current active formal-sector employment by department/Callao. Peru, ENDES 2005–2023. Panel **(A)**: choropleth map of weighted EBF prevalence by political-administrative unit. Panel **(B)**: choropleth map of the proportion of current active formal-sector employment among mothers of children younger than six months. Visual comparison of both maps shows an inverse aggregate pattern; the pattern should be interpreted at the territorial level.

EBF prevalence varied across the 25 political-administrative units evaluated (Figure 2). The highest prevalences were recorded in Puno, Huancavelica, Apurimac, and Ayacucho, with values above 80%, whereas several coastal units had prevalences below 65%. The proportion of maternal current active formal-sector employment was higher in Lima, Moquegua, Arequipa, Ica, Callao, and Tacna, whereas in several units of the Highlands and Amazon it was below 5%.

**Figure 3:**
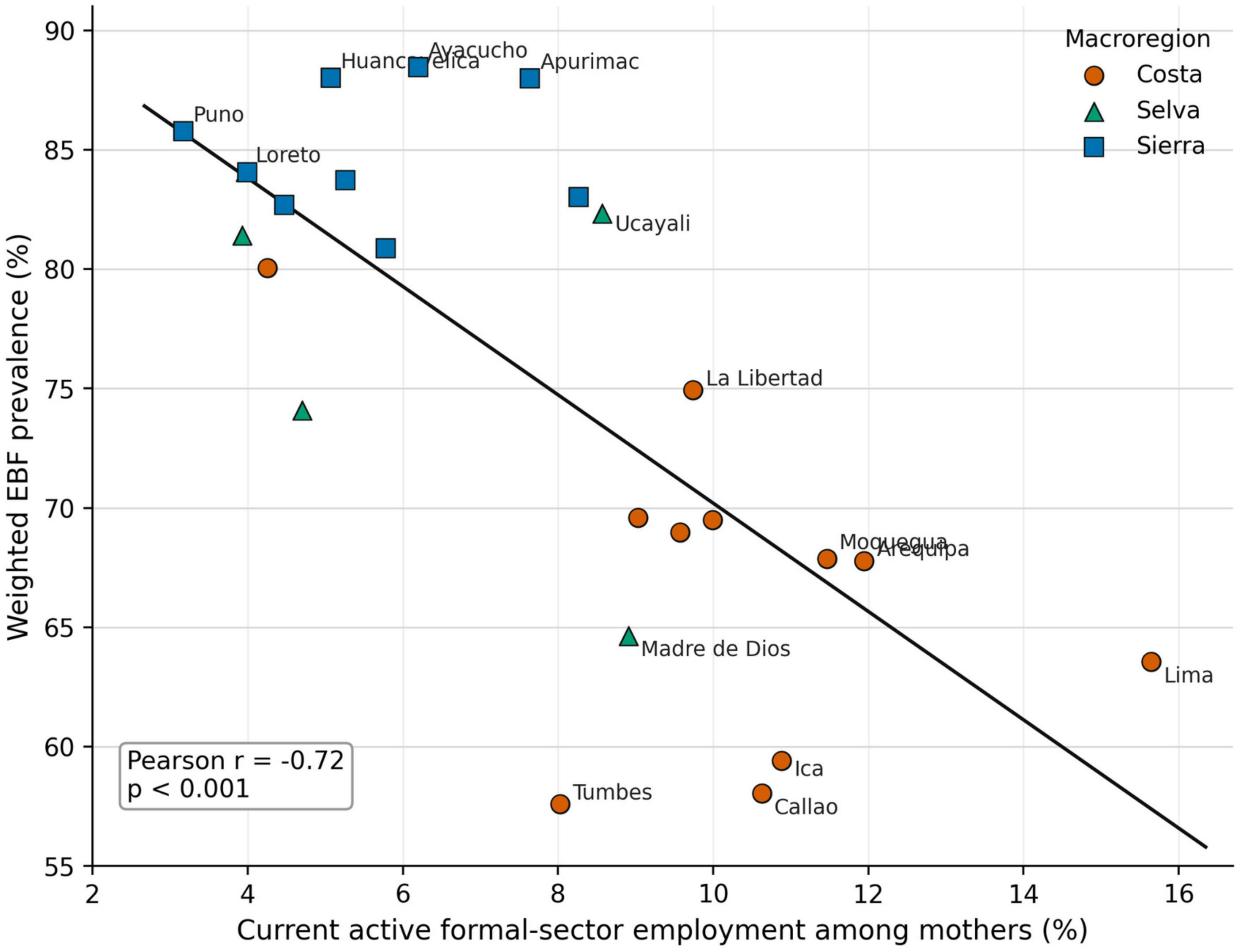
Correlation between the proportion of maternal current active formal-sector employment and exclusive breastfeeding prevalence at the departmental/Callao level. Peru, ENDES 2005–2023. Scatter plot showing the ecological association between the proportion of maternal current active formal-sector employment (*x*-axis) and EBF prevalence (*y*-axis) at the aggregate level. Each point represents a political-administrative unit and uses grayscale-compatible symbols by macroregion. The diagonal line represents the ecological linear regression. This figure is hypothesis-generating and should not be interpreted as evidence of individual-level association.

In the ecological analysis at the territorial level, an inverse relationship was observed between the proportion of maternal current active formal-sector employment and EBF prevalence (Figure 3). Territories with a higher proportion of maternal current active formal-sector employment tended to have lower EBF prevalences. Pearson's correlation coefficient was *r* = −0.72 (*p* < 0.001). This pattern was interpreted as contextual and hypothesis-generating, not as corroboration of the individual-level association.

### 3.8 Interaction analysis between current active formal-sector employment, socioeconomic level, and residence area

Exploratory additive-interaction analyses did not show conclusive evidence that the disparity associated with current active formal-sector employment differed additively by high socioeconomic level or urban residence. Because EBF is a favorable outcome and interaction estimates were imprecise, these analyses were interpreted only as exploratory assessments of heterogeneity (Supplementary Table S3).

## 4 Discussion

### 4.1 Main findings

In this pooled cross-sectional analysis of ENDES 2005–2023, current active formal-sector employment was associated with substantially lower EBF prevalence than no current survey-defined employment. This disparity persisted after adjustment for maternal, child, household, territorial, and survey-year covariates, and sensitivity analyses including all mothers regardless of marital status yielded similar results. Because the exposure captures active formal-sector work at the time of interview rather than the mere existence of a formal employment relationship, this finding is better interpreted as a disparity linked to postpartum participation in formal-sector work, not as evidence that labor formality itself reduces breastfeeding.

### 4.2 Comparison with the literature and interpretation of findings

The results are consistent with international evidence recognizing breastfeeding as a practice shaped by social, economic, regulatory, and labor-related determinants rather than as an exclusively individual behavior. The lower EBF prevalence observed among mothers with current active formal-sector employment should not be interpreted as definitive evidence of structural inequality or as proof that labor formality is harmful. A more cautious interpretation is that this category may capture a combination of early postpartum return to formal-sector work, workplace schedules, commuting time, urban living conditions, higher socioeconomic position, health-promotion gaps, and regional feeding norms that may be less compatible with continued EBF.

In Peru, previous ENDES-based studies have described socioeconomic and territorial inequalities in EBF, with this practice more concentrated among lower-wealth households and residents of rural areas, the Highlands, and the Amazon region (10). Our revised analysis reproduced this pattern: EBF was more frequent in rural areas, in the Highlands and Amazon, and in the lowest wealth quintiles, and less frequent in Metropolitan Lima, urban areas, and the highest wealth quintile. Current active formal-sector employment was concentrated mainly in urban contexts and in more advantaged socioeconomic groups, which supports the need to interpret the employment disparity together with broader urban and socioeconomic patterning.

The association between maternal employment and lower breastfeeding continuity has been documented in different contexts. In low- and middle-income countries, the duration of maternity leave has been associated with better breastfeeding practices, including exclusive breastfeeding, supporting the importance of protective labor policies during the postpartum period (7). Similarly, studies and reviews among working mothers have shown that returning to work may represent a critical point for breastfeeding interruption or reduction, especially when working conditions do not facilitate the expression, storage, and administration of breast milk (6, 8). A study conducted in Kenya reported that formal maternal employment was associated with lower odds of exclusive breastfeeding in the early postpartum period, a finding consistent with the direction of the association observed in our analysis (16).

Urban settings may also offer resources that could facilitate expressed-milk feeding, including better access to electricity, refrigeration, health services, and breast-milk expression equipment. Therefore, the lower EBF prevalence observed in urban and higher-wealth groups should not be interpreted as a simple lack-of-resources phenomenon. It may instead reflect opportunity costs, workplace demands, commuting, feeding preferences, and social norms that operate despite greater material access.

The additive interaction analysis did not show conclusive evidence of synergy between current active formal-sector employment and socioeconomic level or residence area. This result suggests that the observed gap was not clearly concentrated within only one socioeconomic or urban subgroup. Nevertheless, the absence of statistical interaction does not rule out heterogeneity in underlying mechanisms, because barriers may differ between higher-income urban workers and lower-income or peri-urban workers who remain within formal-sector arrangements.

The territorial analysis provides a contextual, hypothesis-generating pattern. Territories with a higher proportion of maternal current active formal-sector employment, particularly Lima, Callao, and several coastal regions, had lower EBF prevalences, whereas Andean and Amazonian departments recorded higher prevalences. However, aggregate analyses compare territorial units and do not allow direct inference about individual associations because urbanization, wealth, healthcare access, and cultural norms cluster geographically (17). Therefore, this ecological pattern should not be interpreted as corroborating the individual-level association.

The descriptive analysis of reasons for cessation provides a complementary but limited signal. The reason “work” was reported more frequently by mothers with current active formal-sector employment than by other groups, which is consistent with evidence identifying return to work as a critical moment for breastfeeding continuity (8). In contrast, “no breast milk” was kept separate because it may reflect perceived milk insufficiency, physiological concerns, counselling gaps, or social beliefs rather than a direct occupational constraint. These data therefore suggest plausible mechanisms but do not establish temporality or causation.

### 4.3 Implications for public health and maternal and child health

From a public health perspective, the findings identify mothers with current active formal-sector employment as a group for further evaluation of employment-compatible breastfeeding support. The results do not directly evaluate specific workplace or health policies. Rather, they indicate that workplace, health-service, and household conditions should be studied jointly to understand why a group with greater formal-sector attachment and material resources may still experience lower EBF prevalence.

International evidence supports that maternity-leave policies and workplace interventions can favor breastfeeding continuity when effectively implemented (6–8). In Peru, maternity and breastfeeding-related workplace protections include 49 days of prenatal leave and 49 days of postnatal leave, breastfeeding breaks after return to work until the child reaches one year of age, and institutional lactation rooms in public- and private-sector workplaces (18–21). However, ENDES does not measure effective leave duration, timing of return to work, actual use of breastfeeding breaks, lactation-room availability, supervisor support, workplace culture, or regulatory compliance. These legal and organizational factors should therefore be interpreted as plausible contextual mechanisms requiring direct evaluation, not as mechanisms demonstrated by the present analysis.

These findings should also be interpreted in relation to the broader maternity-protection framework, which recognizes paid maternity leave, breastfeeding breaks, and breastfeeding facilities as relevant conditions for breastfeeding continuation (22). This entails preparing individualized breastfeeding-continuity plans, discussing milk expression and storage, providing guidance on labor rights related to breastfeeding, and coordinating consistent messages among health facilities, employers, and families. Qualitative and mixed-methods studies are needed to understand mothers' lived experiences, workplace acceptability of breastfeeding support, and the reasons why greater material access in urban and higher-wealth groups may not translate into higher EBF prevalence.

From a programmatic perspective, monitoring only the national prevalence of EBF is insufficient. Surveillance should also track gaps by current maternal employment status, residence area, region, and socioeconomic level. In the formal sector, future evaluations should measure implementation and use of lactation rooms, effective access to breastfeeding breaks, barriers reported by workers, employer practices, and coordination between occupational health and maternal and child health.

Accordingly, reducing this disparity should be considered part of a broader agenda of maternal and child health equity, social protection, and shared responsibility for care. The key empirical task is no longer to assume that regulations work or fail, but to evaluate their enforcement, real accessibility, acceptability to workers, and capacity to sustain EBF during the first six months of life.

### 4.4 Study limitations

These findings should be interpreted in light of several limitations. First, current maternal employment status was approximated using survey information on current work and employer type, which does not capture contracts, tax registration, pension contributions, job stability, or effective compliance with labor rights. This may have led to misclassification, particularly among self-employed women operating formally and among women with formal jobs who were temporarily away from work because of maternity leave. Second, the cross-sectional design and 24 h recall measurement of EBF preclude inference about breastfeeding continuity from birth to six months and do not establish temporality between employment conditions and EBF. Third, residual confounding is possible because some breastfeeding-related variables, including breastfeeding counselling, were not included in the primary model or were not consistently harmonized across all survey years. Delivery mode and place of delivery were evaluated in a sensitivity analysis and did not materially change the estimates. Fourth, restriction to mothers currently married or cohabiting limits external validity; however, the sensitivity analysis including all mothers produced similar results. Fifth, complete-case analysis may have introduced selection bias if missingness was related to both employment and breastfeeding, although missingness in the main adjusted model was concentrated mainly in maternal age, parity, and a very small number of employment classifications. Finally, temporal and ecological analyses were exploratory and should be interpreted as contextual descriptions rather than causal evaluations of policies, workplace conditions, or territorial determinants.

## 5 Conclusions

In Peru, mothers currently married or cohabiting and classified as having current active formal-sector employment had a lower prevalence of EBF during 2005–2023. Because this exposure reflects active formal-sector work at the time of survey rather than administrative labor formality itself, the findings should be interpreted as descriptive disparities related to postpartum formal-sector workforce participation. The inverse association persisted after adjustment for major sociodemographic, territorial, and survey-year covariates and was consistent in sensitivity analyses including all mothers.

Future studies should evaluate the mechanisms underlying this gap, including timing of return to work, effective use of maternity leave, breastfeeding breaks, lactation rooms, workplace culture, counselling quality, and mothers' lived experiences. Qualitative and mixed-methods research would be particularly useful to understand why mothers with greater material resources and formal-sector attachment may still experience lower EBF prevalence, and to identify employment-compatible support strategies appropriate for the Peruvian context.

## Data Availability

Publicly available datasets were analyzed in this study. This data can be found here: https://proyectos.inei.gob.pe/microdatos/
