# Supplementary material for: Disparities in exclusive breastfeeding by current maternal employment status in Peru, 2005–2023: a cross-sectional analysis of ENDES data
Source: Front Glob Womens Health. 2026 Jul 20;7:1865825. doi: 10.3389/fgwh.2026.1865825 (PMC13429758; doi:10.3389/fgwh.2026.1865825)
Supplement: Supplementary file 1 [file Table1.docx]

Supplementary Material

**STROBE checklist for cross-sectional studies**

| **No.** | **Recommendation** | **Location in the manuscript** |
| --- | --- | --- |
| TITLE AND ABSTRACT | TITLE AND ABSTRACT | TITLE AND ABSTRACT |
| 1a | Indicate the study design in the title or abstract using a commonly used term. | Title: includes the design ('cross-sectional analysis') and the study period (2005-2023). Abstract, Methods section: analytical cross-sectional study using pooled ENDES data. |
| 1b | Provide an informative and balanced summary in the abstract of what was done and what was found. | Abstract: includes objective, methods (design, source, revised sample restricted to mothers currently married or cohabiting, EBF definition, employment-status classification, and adjusted analysis), main results (overall prevalence 72.6%; current active formal-sector employment adjusted PR=0.72; ecological correlation r=-0.72), and conclusions with descriptive cross-sectional interpretation. |
| INTRODUCTION | INTRODUCTION | INTRODUCTION |
| 2 | Background/rationale: Explain the scientific background and rationale for the investigation being reported. | Introduction, paragraphs 1-4: global burden of EBF (WHO/UNICEF), clinical and public health relevance, structural determinants of breastfeeding (social determinants, working conditions), variations in Latin America and Peru, and evidence gap regarding current maternal employment status and EBF. |
| 3 | Objectives: State the specific objectives, including any prespecified hypotheses. | Introduction, final paragraph: the objective was to estimate descriptive disparities in EBF prevalence according to current maternal employment status among mothers currently married or cohabiting with children younger than six months in Peru, using nineteen ENDES rounds from 2005 to 2023. |
| METHODS | METHODS | METHODS |
| 4 | Study design: Present the key elements of the study design early in the article. | Methods, 'Study design': observational analytical cross-sectional study based on repeated surveys; publicly available ENDES secondary data; 19 annual rounds (2005-2023); analytical objective: estimation of absolute and relative disparities according to current maternal employment status using categories explicitly labelled as no current survey-defined employment, current active formal-sector employment, and current active informal-sector employment. |
| 4* | Note: STROBE item 4 for cross-sectional studies asks authors to describe whether participants were recruited or selected from a source population or from available records. | Methods, 'Data source': secondary data from ENDES, a continuous INEI survey with a complex probabilistic, stratified, two-stage sampling design and national representativeness. |
| 5 | Setting: Describe the setting, locations, and relevant dates, including periods of recruitment, exposure, follow-up, and data collection. | Methods, 'Data source': ENDES conducted by INEI in Peru; 19 annual rounds during 2005-2023; two-stage design with national, urban-rural, natural-region, and departmental representativeness; publicly available data and technical documentation on the INEI portal. |
| 6 | Participants: Give eligibility criteria and the sources and methods of participant selection. | Methods, 'Population and sample': women aged 15-49 years from ENDES 2005-2023 with available birth records; inclusion criteria: child <6 months, information in the breastfeeding module, mother currently married or cohabiting, and sufficient data for the outcome and critical variables. Restricted descriptive sample: 15,780 dyads; employment-classified descriptive sample: 15,773; adjusted model complete-case sample: 15,101. |
| 7 | Variables: Clearly define the outcomes, exposures, predictors, potential confounders, and effect modifiers. Give diagnostic criteria when applicable. | Methods, 'Variables': Outcome: EBF (24-hour recall; infant <6 months receiving no liquids or foods other than breast milk). Exposure: current maternal employment status (no current survey-defined employment / current active formal-sector employment / current active informal-sector employment; based on current work and reported employer type). Covariates: maternal age, educational level, parity, child sex and age, wealth quintile, residence area, natural region, and survey year. |
| 8* | Data sources/measurement: For each variable of interest, indicate data sources and details of assessment or measurement methods. Describe comparability of assessment methods if there is more than one group. | Methods, 'Data source' and 'Variable processing and harmonization': EBF obtained from the ENDES breastfeeding and infant feeding module; employment status from the sociodemographic characteristics module. Harmonization of variables across the 19 rounds to ensure temporal comparability; recoding of categories into homogeneous and mutually exclusive classifications. |
| 9 | Bias: Describe any efforts to address potential sources of bias. | Methods, 'Variables' and Discussion, 'Study limitations': the exposure was defined as an operational proxy based on current work and employer type; it is acknowledged that it does not capture all administrative components of labor formality and that mothers with formal employment on leave could be classified as having no current survey-defined employment. Complete-case analysis and potential residual confounding are discussed. |
| 10 | Study size: Explain how the study size was determined. | Methods, 'Population and sample': sample size was determined by the availability of records in ENDES. Results, 'Sample selection': restricted descriptive sample of 15,780 dyads; employment-classified descriptive sample of 15,773; adjusted model complete-case sample of 15,101. |
| 11 | Quantitative variables: Explain how quantitative variables were handled in the analyses. If applicable, describe the groups used and why. | Methods, 'Variables': maternal age (completed years) categorized as 15-19, 20-34, and 35-49 years; child age (months) grouped as 0-<2, 2-<4, and 4-<6 months for descriptive analysis. Wealth quintile used as an ordinal variable (Q1 to Q5). No continuous transformations were performed; quantitative variables were handled as categorical variables in the main analyses. |
| 12a | Statistical methods: Describe all statistical methods, including those used to control for confounding. | Methods, 'Statistical analysis': survey-weighted prevalences with 95% CIs; survey-weighted Poisson regression with log link and robust standard errors; crude and adjusted PRs. The adjusted model included maternal age, educational level, wealth quintile, parity, child age, child sex, urban/rural residence, natural region, and survey year as a categorical covariate. |
| 12b | Describe methods used to examine subgroups and interactions. | Methods, 'Statistical analysis': exploratory additive interaction analysis between current active formal-sector employment and high socioeconomic level (Q4-Q5), and between current active formal-sector employment and urban residence; RERI, AP, and synergy index with 95% CIs using the delta method. |
| 12c | Explain how missing data were addressed. | Methods, 'Population and sample' and Results, 'Sample selection': complete-case analysis was performed; no missing-data imputations were conducted. Missingness for the adjusted model was concentrated mainly in maternal age (n=523), parity (n=150), and employment classification (n=7). |
| 12d | For cross-sectional studies: if applicable, describe analytical methods that account for the sampling strategy. | Methods, 'Statistical analysis': analyses incorporated the complex sampling design (weights, strata, and primary sampling units) using the Stata 17 survey module. For the pooled analysis of 19 rounds, each year's sampling weight was rescaled by dividing by the number of rounds. |
| 12e | Describe any sensitivity analyses. | Sensitivity analyses included all mothers regardless of marital status with additional adjustment for marital status, and additional adjustment for mode of delivery and place of delivery. Temporal heterogeneity was assessed using employment-status interactions with continuous survey year and prespecified periods. |
| RESULTS | RESULTS | RESULTS |
| 13a | Report the number of individuals at each stage of the study--e.g., numbers potentially eligible, examined for eligibility, confirmed eligible, included in the study, completing follow-up, and analyzed. | Results, 'Sample selection': children <6 months in ENDES 2005-2023 (n=18,882) -> exclusion of mothers not currently married/cohabiting or marital status unavailable (n=2,919) -> currently married/cohabiting before complete-case rule (n=15,963) -> exclusion for critical missing data (n=183) -> restricted descriptive sample (n=15,780) -> employment-classified descriptive sample (n=15,773) -> adjusted model complete-case sample (n=15,101). |
| 13b | Give reasons for non-participation at each stage. | Results, 'Sample selection': reasons for exclusion are specified at each stage (child age criterion, maternal marital status, critical missing information). |
| 13c | Consider use of a flow diagram. | Supplementary Figure S1: flow diagram of the sample selection process. |
| 14a | Give characteristics of study participants (e.g., demographic, clinical, social) and information on exposures and potential confounders. | Results, 'Characteristics of the study population' and Table 1: sociodemographic and child characteristics according to maternal employment type in the restricted descriptive sample (n=15,780), with absolute frequencies and weighted percentages. |
| 14b | Indicate the number of participants with missing data for each variable of interest. | Table 1 footnote and Results, 'Sample selection': missing data are reported for maternal age (n=523), parity (n=150), and employment classification (n=7); the adjusted model complete-case sample included 15,101 dyads. |
| 14c | For cross-sectional studies: indicate the follow-up period if applicable. | Not applicable. The study is cross-sectional; participants were not followed over time. Each dyad was observed at a single time point (interview in the corresponding ENDES round). |
| 15* | For cross-sectional studies: report the number of outcome events or provide summary measures. | Results, 'EBF prevalence according to current maternal employment status': overall weighted EBF prevalence 72.6%; no current survey-defined employment 76.1%, current active informal-sector employment 74.1%, and current active formal-sector employment 46.8% (Supplementary Table S1). |
| 16a | Give unadjusted estimates and, if applicable, estimates adjusted for confounders. If adjusted estimates are calculated, indicate which confounders were included and why. | Table 2: crude and adjusted PRs with 95% CIs for current active formal-sector employment (crude PR=0.61; 0.55-0.67; adjusted PR=0.72; 0.65-0.79) and current active informal-sector employment (crude PR=0.97; 0.94-1.01; adjusted PR=0.97; 0.94-1.00), using no current survey-defined employment as the reference. |
| 16b | Report other analyses performed (e.g., subgroup and interaction analyses, and sensitivity analyses). | Results: weighted temporal trend and absolute gap analyses (Figure 1); sensitivity including all mothers (Supplementary Table S4); period-specific adjusted PRs (Supplementary Table S5); delivery-covariate sensitivity analysis (Supplementary Table S6); reasons for breastfeeding cessation (Supplementary Table S2); territorial distribution and ecological correlation (Figures 2 and 3); additive interaction analysis (Supplementary Table S3). |
| DISCUSSION | DISCUSSION | DISCUSSION |
| 17 | Key results: Summarize key results with reference to study objectives. | Discussion, 'Main findings': lower EBF prevalence among mothers with current active formal-sector employment compared with mothers with no current survey-defined employment; adjusted and sensitivity results; persistence of the disparity across periods; descriptive cross-sectional interpretation. |
| 18 | Limitations: Discuss limitations of the study, considering sources of potential bias or imprecision. Discuss the direction and magnitude of any potential bias. | Discussion, 'Study limitations': proxy measurement of current employment status/formality; possible classification of mothers on maternity leave as no current survey-defined employment; absence of direct measures of leave duration, return-to-work timing, use of breastfeeding breaks, lactation rooms, supervisor support, and counselling; 24-hour recall; primary restriction to mothers currently married or cohabiting; complete-case analysis; ecological and temporal analyses interpreted descriptively. |
| 19 | Interpretation: Provide a cautious overall interpretation of results considering objectives, limitations, multiplicity of analyses, results from similar studies, and other relevant evidence. | Discussion, 'Comparison with the literature and interpretation of findings': lower EBF prevalence among mothers with current active formal-sector employment is interpreted as a descriptive disparity linked to postpartum formal-sector workforce participation, not as a causal effect of labor formality. The text discusses urban/socioeconomic patterning, return to work, maternity leave, workplace support, and ecological limitations. |
| 20 | Generalizability: Discuss the generalizability (external validity) of the study results. | Discussion, 'Study limitations': inference from the primary analysis corresponds to mothers currently married or cohabiting with infants <6 months; sensitivity analysis including all mothers was added to assess robustness and yielded similar estimates. |
| OTHER INFORMATION | OTHER INFORMATION | OTHER INFORMATION |
| 21 | Funding: State the study funding and the role of funders, and, if applicable, for the original study. | Declarations, 'Funding': article processing charges will be covered by the Vice-Rectorate for Research of UNTRM; it is explicitly stated that the funder had no role in design, analysis, interpretation, publication decision, or manuscript preparation. |

** Items marked with an asterisk apply specifically to studies with more than one group or with measurement of several variables. EBF = exclusive breastfeeding; ENDES = Demographic and Family Health Survey; INEI = National Institute of Statistics and Informatics; PR = prevalence ratio; 95% CI = 95% confidence interval; RERI = relative excess risk due to interaction; AP = attributable proportion; UNTRM = Universidad Nacional Toribio Rodríguez de Mendoza de Amazonas.*

**Supplementary Table S1. Weighted prevalence of exclusive breastfeeding according to maternal, child, and household characteristics. Peru, ENDES 2005-2023.**

| **Characteristic** | **n/N** | **Prevalence (%)** | **95% CI** |
| --- | --- | --- | --- |
| Total | 11,815/15,780 | 72.6 | 71.5-73.7 |
| **Current maternal employment status** |  |  |  |
| No current survey-defined employment | 7,325/9,423 | 76.1 | 74.7-77.4 |
| Current active formal-sector employment | 624/1,259 | 46.8 | 42.5-51.1 |
| Current active informal-sector employment | 3,861/5,091 | 74.1 | 72.2-76.0 |
| **Maternal age** |  |  |  |
| 15-19 years | 1,788/2,272 | 75.7 | 72.9-78.5 |
| 20-34 years | 7,646/10,156 | 73.3 | 71.9-74.6 |
| 35-49 years | 2,016/2,829 | 68.9 | 66.1-71.6 |
| **Educational level** |  |  |  |
| No education/initial | 4,191/5,519 | 74.0 | 72.1-75.8 |
| Primary | 2,871/3,625 | 77.5 | 75.5-79.5 |
| Secondary | 3,314/4,419 | 73.3 | 71.4-75.3 |
| Higher education | 1,438/2,216 | 62.1 | 58.8-65.3 |
| **Parity** |  |  |  |
| Primiparous | 2,130/3,148 | 65.0 | 62.4-67.6 |
| 2-3 children | 6,090/8,176 | 72.9 | 71.4-74.5 |
| 4 or more children | 3,478/4,306 | 78.7 | 76.9-80.6 |
| **Child sex** |  |  |  |
| Male | 6,062/8,084 | 72.3 | 70.7-73.9 |
| Female | 5,753/7,696 | 73.0 | 71.5-74.5 |
| **Child age** |  |  |  |
| 0-<2 months | 2,927/3,604 | 79.5 | 77.4-81.5 |
| 2-<4 months | 4,442/5,660 | 75.1 | 73.2-76.9 |
| 4-<6 months | 4,446/6,516 | 66.9 | 65.1-68.7 |
| **Wealth quintile** |  |  |  |
| Q1 (poorest) | 4,146/4,774 | 86.6 | 85.2-88.0 |
| Q2 | 3,326/4,349 | 77.4 | 75.5-79.3 |
| Q3 | 2,215/3,085 | 72.1 | 69.7-74.4 |
| Q4 | 1,367/2,134 | 66.8 | 63.8-69.9 |
| Q5 (richest) | 761/1,438 | 51.1 | 47.3-54.8 |
| **Residence area** |  |  |  |
| Urban | 6,760/9,810 | 67.5 | 66.0-68.9 |
| Rural | 5,055/5,970 | 84.9 | 83.7-86.2 |
| **Natural region** |  |  |  |
| Metropolitan Lima | 947/1,521 | 62.9 | 59.9-65.9 |
| Rest of Coast | 2,773/4,246 | 67.3 | 65.4-69.2 |
| Highlands | 4,615/5,525 | 82.2 | 80.8-83.6 |
| Amazon | 3,480/4,488 | 80.3 | 78.7-82.0 |

*Prevalences and 95% CIs estimated using svy: proportion with logit intervals. n/N = EBF cases/total by category (unweighted absolute frequencies).*

**Supplementary Table S2. Exploratory analysis: reasons for breastfeeding cessation reported by mothers, according to current maternal employment status. Peru, ENDES 2005-2023.**

| **Reason for cessation** | **Total** | **No current survey-defined employment** | **Current active formal-sector employment** | **Current active informal-sector employment** |
| --- | --- | --- | --- | --- |
| Weaning age | 1,433 (31.6) | 877 (31.8) | 87 (26.3) | 469 (33.2) |
| Became pregnant | 914 (24.3) | 546 (24.4) | 45 (16.7) | 323 (26.9) |
| Insufficient breast milk | 496 (10.6) | 336 (12.3) | 12 (3.2) | 148 (9.8) |
| Other health reason | 345 (8.9) | 214 (9.3) | 22 (8.8) | 109 (8.3) |
| Work | 278 (8.1) | 140 (6.5) | 52 (18.1) | 86 (7.5) |
| No breast milk | 261 (8.0) | 137 (7.0) | 49 (17.8) | 75 (6.3) |
| Child refused | 136 (4.4) | 86 (4.2) | 10 (5.2) | 40 (4.3) |
| Child was old enough | 69 (1.7) | 49 (1.9) | 2 (1.2) | 18 (1.5) |
| Child illness | 56 (1.0) | 41 (1.3) | 2 (0.6) | 13 (0.5) |
| Maternal illness | 32 (0.6) | 20 (0.7) | 7 (0.5) | 5 (0.5) |
| Nipple problem | 24 (0.7) | 13 (0.4) | 5 (1.5) | 6 (1.1) |
| Other reason | 5 (0.1) | 4 (0.1) | 0 (0.0) | 1 (0.3) |

*Exploratory and descriptive analysis. Values are expressed as n (weighted %). Percentages were weighted for the complex sampling design. Forty-six observations with code 96 (does not know/does not remember) were excluded. The categories "Work" and "No breast milk" are presented separately because they represent different constructs: occupational constraints versus perceived milk insufficiency, physiological concerns, counselling gaps, or social beliefs. They were not combined into a single indicator.*

**Supplementary Table S3. Exploratory additive interaction analysis (RERI) between current active formal-sector employment and socioeconomic level/residence area. Peru, ENDES 2005-2023.**

| **Measure** | **Estimate** | **95% CI** |
| --- | --- | --- |
| **Current active formal-sector employment x Rich quintile (Q4-Q5)** |  |  |
| EBF prev.: No current active formal-sector employment / Not rich | 77.9% | 76.5 - 79.2 |
| EBF prev.: No current active formal-sector employment / Rich | 63.4% | 60.2 - 66.4 |
| EBF prev.: Current active formal-sector / Not rich | 53.1% | 45.7 - 60.3 |
| EBF prev.: Current active formal-sector / Rich | 46.6% | 41.2 - 52.1 |
| PR: No current active formal-sector employment / Rich vs ref. | 0.81 | 0.77 - 0.86 |
| PR: Current active formal-sector / Not rich vs ref. | 0.68 | 0.59 - 0.78 |
| PR: Current active formal-sector / Rich vs ref. | 0.60 | 0.53 - 0.67 |
| RERI | 0.103 | -0.020 - 0.227 |
| AP | 0.173 | -0.024 - 0.369 |
| S (synergy index) | 0.795 | 0.584 - 1.007 |
| **Current active formal-sector employment x Urban area** |  |  |
| RERI | 0.048 | -0.113 - 0.208 |
| AP | 0.085 | -0.200 - 0.370 |
| S (synergy index) | 0.903 | 0.602 - 1.203 |

*Exploratory analysis. RERI = relative excess risk due to interaction; AP = attributable proportion due to interaction; S = synergy index. Because EBF is a favorable outcome, interaction measures should be interpreted with caution and not as direct evidence of adverse synergy. Prevalences and 95% CIs were obtained from svy: proportion. PRs and interaction measures were estimated using Poisson regression with robust variance. RERI CIs were calculated with nlcom in Stata. Overall, the CIs for the main measures do not show conclusive evidence of additive interaction.*

**Supplementary Table S4. Sensitivity analysis including all mothers with infants younger than six months. Peru, ENDES 2005-2023.**

| **Current maternal employment status** | **n/N** | **EBF prevalence (%; 95% CI)** | **Crude PR (95% CI)** | **Adjusted PR (95% CI)** |
| --- | --- | --- | --- | --- |
| No current survey-defined employment | 7,677/9,919 | 75.6 (74.3-76.9) | Reference | Reference |
| Current active formal-sector employment | 704/1,417 | 48.4 (44.3-52.5) | 0.64 (0.59-0.70) | 0.74 (0.68-0.81) |
| Current active informal-sector employment | 4,470/5,883 | 74.1 (72.3-75.9) | 0.98 (0.95-1.01) | 0.97 (0.94-1.00) |

*This model included all mothers with infants younger than six months and additionally adjusted for marital status. Adjusted models also included maternal age, educational level, wealth quintile, parity, child age, child sex, urban/rural residence, natural region, and survey year.*

**Supplementary Table S5. Period-specific adjusted prevalence ratios for exclusive breastfeeding according to current maternal employment status. Peru, ENDES 2005-2023.**

| **Period** | **Current active formal-sector employment vs. no current survey-defined employment** | **Current active informal-sector employment vs. no current survey-defined employment** |
| --- | --- | --- |
| 2005-2012 | 0.68 (0.56-0.83) | 1.02 (0.97-1.07) |
| 2013-2019 | 0.73 (0.64-0.84) | 0.95 (0.91-0.99) |
| 2020-2021 | 0.63 (0.45-0.89) | 1.02 (0.91-1.13) |
| 2022-2023 | 0.76 (0.62-0.93) | 0.86 (0.77-0.96) |

*Period-specific adjusted PRs were estimated using the same covariate set as the main adjusted model, excluding survey year when period was used as the stratifying variable. Interaction tests: employment status x continuous survey year, p=0.041; employment status x prespecified period, p=0.058.*

**Supplementary Table S6. Sensitivity analysis additionally adjusting for mode of delivery and place of delivery. Peru, ENDES 2005-2023.**

| **Model** | **Employment group** | **n** | **Adjusted PR (95% CI)** |
| --- | --- | --- | --- |
| Base adjusted model | Current active formal-sector employment | 1,216 | 0.72 (0.65-0.79) |
| Base adjusted model | Current active informal-sector employment | 4,869 | 0.97 (0.94-1.00) |
| Plus mode of delivery and place of delivery | Current active formal-sector employment | 1,213 | 0.73 (0.66-0.81) |
| Plus mode of delivery and place of delivery | Current active informal-sector employment | 4,812 | 0.97 (0.94-1.00) |

*The delivery-covariate sensitivity analysis included caesarean delivery and place of delivery. Breastfeeding-counselling variables were not included because comparable information was not available across all 2005-2023 survey rounds and missingness followed a year-dependent pattern.*

**Supplementary Figure S1. Sample selection flow diagram.**


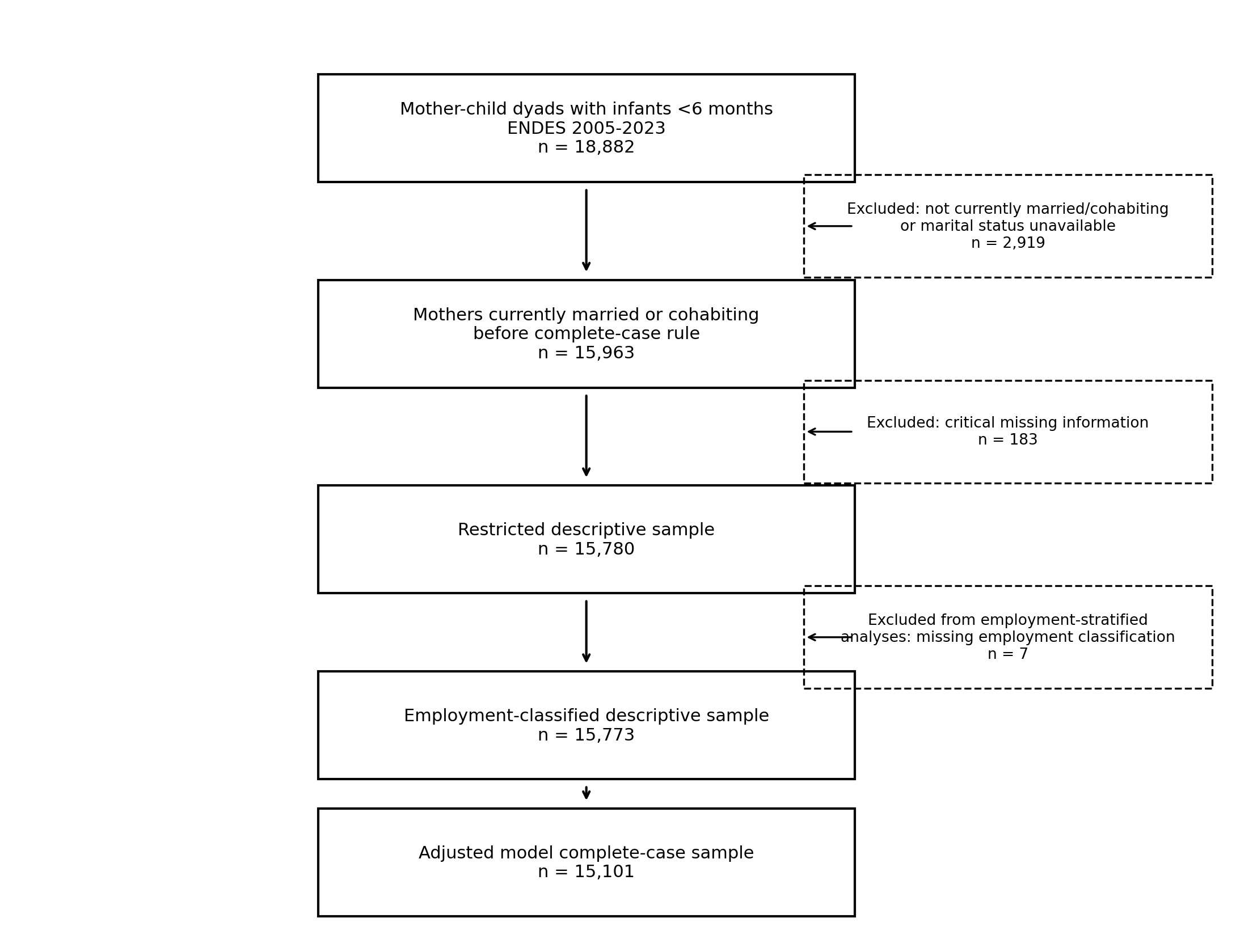


*Note: estimates stratified by current maternal employment status excluded 7 records without a valid employment classification. The adjusted model complete-case sample included 15,101 dyads.*
